# The cross-metathesis of methyl oleate with *cis*-2-butene-1,4-diyl diacetate and the influence of protecting groups

**DOI:** 10.3762/bjoc.7.1

**Published:** 2011-01-03

**Authors:** Arno Behr, Jessica Pérez Gomes

**Affiliations:** 1 Chair of Technical Chemistry A, Department of Biochemical and Chemical Engineering, Technische Universität Dortmund, Emil-Figge-Str. 66, D-44227 Dortmund, Germany

**Keywords:** cross-metathesis, methyl oleate, monomer, polyester, renewable resources

## Abstract

**Background: ***α*,*ω*-Difunctional substrates are useful intermediates for polymer synthesis. An attractive, sustainable and selective (but as yet unused) method in the chemical industry is the oleochemical cross-metathesis with preferably symmetric functionalised substrates. The current study explores the cross-metathesis of methyl oleate (**1**) with *cis*-2-butene-1,4-diyl diacetate (**2**) starting from renewable resources and quite inexpensive base chemicals.

**Results:** This cross-metathesis reaction was carried out with several phosphine and *N*-heterocyclic carbene ruthenium catalysts. The reaction conditions were optimised for high conversions in combination with high cross-metathesis selectivity. The influence of protecting groups present in the substrates on the necessary catalyst loading was also investigated.

**Conclusions:** The value-added methyl 11-acetoxyundec-9-enoate (**3**) and undec-2-enyl acetate (**4**) are accessed with nearly quantitative oleochemical conversions and high cross-metathesis selectivity under mild reaction conditions. These two cross-metathesis products can be potentially used as functional monomers for diverse sustainable polymers.

## Introduction

In the last decade, olefin metathesis has become a routine and competent synthetic method for the formation of carbon–carbon double bonds [1–5]. Among investigations of ring opening metathesis polymerisation [6] and ring closing metathesis [7], the olefin cross-metathesis has demonstrated its great importance in providing access to alkenes bearing a wide range of functional groups [8–11]. Especially, the olefin cross-metathesis with oleochemicals offers a versatile synthetic approach to prepare value-added substrates starting from renewable raw materials. Due to the cross-metathesis reactions of fatty acid derivatives that yield diverse types of *α,ω*-difunctional monomers, which can be processed into polymers (polyamides, polyesters, polyolefins, etc.), partial or even complete substitution of the steadily decreasing petrochemicals by materials from renewable resources is warranted [12–14]. So far, cross-metathesis reactions of these raw materials with different cross-metathesis reaction partners (allyl alcohol [15–16], allyl chloride [16], acrylonitrile [17], fumaronitrile [18], acrolein [19], methyl acrylate [20] and diethyl maleate [21]) yielding *α,ω*-difunctional substrates have been investigated.

**Scheme 1:**
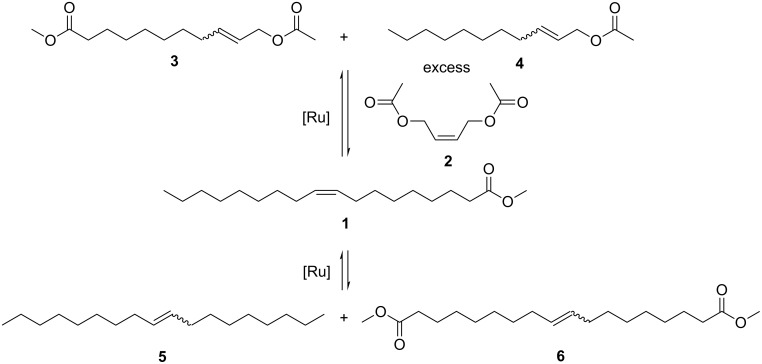
Cross-metathesis of methyl oleate (**1**) with *cis*-2-butene-1,4-diyl diacetate (**2**) and the self-metathesis of **1**.

In this article, the ruthenium catalysed cross-metathesis of methyl oleate (**1**) with *cis*-2-butene-1,4-diyl diacetate (**2**) (Scheme 1) will be described. This synthetic approach gives rise to another group of *α*,*ω*-difunctional substrates: The metathetical conversion studied yields methyl 11-acetoxyundec-9-enoate (**3**) and undec-2-enyl acetate (**4**). The resulting protected *α*-hydroxy-*ω*-carboxylic acid derivatives have potential applications in the preparation of a variety of polymers [22] or lactones [14]. Moreover, undec-2-enyl acetate (**4**) could be processed into polyallylic alcohols under appropriate reaction conditions [22]. In contrast to many other oleochemical cross-metathesis reactions, both the resulting products can be used in polymer chemistry. This oleochemical cross-metathesis reaction described here was studied under different reaction conditions with the aim of optimising oleochemical conversions in combination with high cross-metathesis selectivities. Additionally, several phosphine and *N*-heterocyclic carbene ruthenium catalysts were studied. The optimised reaction conditions were subsequently investigated in the cross-metathesis reaction of oleic acid (**7**) with the unprotected *cis*-2-butene-1,4-diol (**8**). By avoiding the use of protecting groups the processing steps to the polymeric end products would be decisively shortened.

Moreover, the advantage of this cross-metathesis reaction is the use of the relatively inexpensive substrates **1** and **2**. The acylated substrate **2** can be directly synthesised by the catalytic reaction of 1,3-butadiene with acetic acid on a large scale. The classical preparative method for 1,4-butanediol is the copper catalysed reaction of acetylene with formaldehyde and subsequent hydrogenation of the intermediate [23].

Currently, the symmetric acylated substrate **2** is one of the most frequently used cross-metathesis substrates in classical metathesis research. For instance, the cross-metathesis of **2** with allyl benzene or terminal aliphatic alkenes has often been used in the development of new metathesis catalysts [24–27]. In oleochemical metathesis research only the tungsten catalysed cross-metathesis of methyl 10-undecenoate with **2** has been described [28]. The highest yield of the resulting cross-metathesis product was 51% after 2 h at 125 °C. With Grubbs 1st generation catalyst **[Ru]-1**, the reaction temperature could be lowered to 45 °C. Quantitative conversions of methyl 10-undecenoate were obtained with a catalyst loading of 5 mol % **[Ru]-1** [8].

## Results and Discussion

Scheme 1 outlines the reaction investigated, i.e., the ruthenium catalysed cross-metathesis of methyl oleate (**1**) with *cis*-2-butene-1,4-diyl diacetate (**2**). The self-metathesis of methyl oleate (**1**), which yields octadec-9-ene (**5**) and dimethyl octadec-9-enedioate (**6**), is the only concurrent metathesis reaction which had to be suppressed (Scheme 1). The desired cross-metathesis products are the *α*,*ω*-diester methyl 11-acetoxyundec-9-enoate (**3**) and undec-2-enyl acetate (**4**). Both are thus derived from a renewable precursor and are interesting substrates for the synthesis of different types of polymers. Substrate **2** can be considered as an important cross-metathesis partner for the metathetical conversion of methyl oleate **1** in terms of its short chain length. Thus, the chain length of the cross-metathesis products is appropriate for certain polymer applications. Therefore, in contrast to polymers prepared from short-chain monomers, these polymers have a higher flexibility and higher stability against hydrolysis [22]. Due to the *cis*-configuration of **2**, the metathesis reactivity is quite high, although this is slightly reduced by the two electron-withdrawing functional groups at the *β*-positions of the double bond. During our investigations of oleochemical cross-metathesis with diethyl maleate, it was found that the *trans*-isomer is less reactive than its *cis*-isomer [21]. The *trans*-isomer is not able to form the metallacyclobutane complex to give the desired metathesis products [28]. In the case of *trans*-2-butene-1,4-diyl diacetate (**2**), the generation of this intermediate cyclic complex is also hindered. In addition, its symmetry only leads to the two desired products.

**Figure 1:**
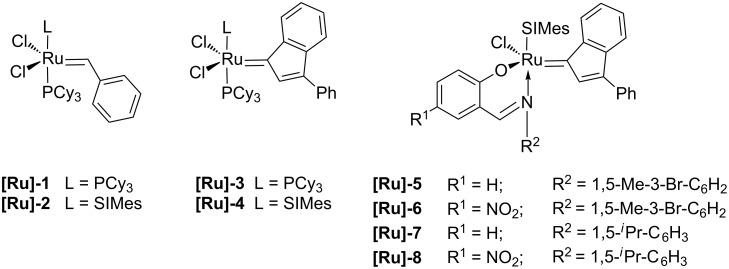
The ruthenium metathesis catalysts used. (SIMes: 1,3-bis-(2,4,6-trimethylphenyl)-4,5-dihydroimidazol-2-ylidene).

The catalytic activity of the ruthenium complexes **[Ru]-1** to **[Ru]-8** (Figure 1) using a catalyst loading of 1.0 mol % was evaluated in the cross-metathesis of methyl oleate (**1**) with *cis*-2-butene-1,4-diyl diacetate (**2**). The reactions were performed with a fivefold excess of **2** in toluene at 50 °C for 5 h to shift the reaction equilibrium towards the cross-metathesis products **3** and **4**.

The differences in metathesis activity of these investigated metathesis initiators are presented in Table 1. Both conversions of **1** and yields of the cross-metathesis products **3** and **4** were determined by gas chromatography with internal standard.

**Table 1:** Results of metathesis catalyst activities in the cross-metathesis of methyl oleate (**1**) with **2**.^a^

entry	catalyst	conversion **1** [%]^b^	yield **3** [%]^b^	yield **4** [%]^b^	yield **5** [%]^b^	yield **6** [%]^b^

**1**	**[Ru]-1**	14	3	4	6	5
**2**	**[Ru]-2**	48	29	28	10	10

**3**	**[Ru]-3**	15	4	4	6	6
**4**	**[Ru]-4**	42	24	26	9	10

**5**	**[Ru]-5**^c^	34	15	14	10	11
**6**	**[Ru]-6**^c^	35	16	15	9	10
**7**	**[Ru]-7**^c^	90	59	58	16	15
**8**	**[Ru]-8**^c^	84	53	52	16	16

^a^reaction conditions: 1.0 mol % cat., *m*(**1**) = 0.17 mmol, 0.85 mmol **2**, toluene, 5 h, 50 °C, 900 rpm; ^b^determined by gas chromatography with internal standard; ^c^addition of 100 equiv of PhSiCl_3_ according to **[Ru]-5**–**[Ru]-8**.

As summarised in Table 1, the lowest conversions of **1** (about 15%) and yields of each of the desired cross-metathesis products **3** and **4** (about 5%) were achieved using the ruthenium phosphine complexes **[Ru]-1** and **[Ru]-3** (Table 1, entries 1 and 3). Only slight differences in activity were observed between the benzylidene catalyst **[Ru]-1** and its indenylidene counterpart **[Ru]-3**. The self-metathesis of methyl oleate (**1**) mentioned above could not be suppressed. Other side-reactions were not observed. No double-bound isomerisation took place. Promising results were obtained with catalysts bearing *N*-heterocyclic carbene ligands (Table 1, entries 2 and 4–8) [29]. Up to 48% of methyl oleate (**1**) was converted and the yields of the cross-metathesis products **3** and **4** of ca. 28% were achievable with ruthenium catalysts **[Ru]-2** and **[Ru]-4** (Table 1, entries 2 and 4). Here, the self-metathesis reaction of **1** is a side-reaction. The yields of **5** and **6** were nearly halved using the second generation ruthenium catalysts **[Ru]-2** and **[Ru]-4**. The cross-metathesis selectivity increased considerable. The difunctionalised co-substrate **2** was also converted to a greater extent. Accordingly, these metathesis catalysts illustrate once more their higher metathesis activity and their higher tolerance towards functional groups [2].

Comparable or higher conversions of **1** and cross-metathesis yields of **3** and **4** (Table 1, entries 5*–*8) were obtained with ruthenium complexes **[Ru]-5**–**[Ru]-8**. Due to their bidentate Schiff base ligands, they must be chemically activated by the addition of 100 equiv of phenyltrichlorosilane [30–31]. Within this catalyst family it can be concluded that with higher steric hindrance of the Schiff base ligands higher conversions and yields were achievable (Table 1, entries 7 and 8). Schiff base ligands with a nitro substituent did not lead to a significant increase or loss of metathesis activity. Consequently, in the oleochemical cross-metathesis reaction **[Ru]-7** and **[Ru]-8** were the most active catalysts due to their space-filling isopropyl substituted Schiff base ligands [31]. The cross-metathesis yields amounted to 59% and methyl oleate (**1**) was converted up to 90%. Moreover, the self-metathesis of methyl oleate (**1**) was reasonably well suppressed and could be considered as a side-reaction; the yield of the self-metathesis products **5** and **6** amounted to 16%. Furthermore, the cross-metathesis selectivity using the ruthenium catalysts **[Ru]-7** and **[Ru]-8** is decisively higher compared to the other catalysts used.

From the results in Table 2 an increase of conversion of methyl oleate **1** (from 15 to 94%) and of yield of each of the cross-metathesis products **3** and **4** (from 2 to 66%) were obtained with a catalyst loading of **[Ru]-7** in a range of 0.1 and 1.5 mol %. Cross-metathesis selectivity was increased by 60% to 67%. With a catalyst loading of 1.5 mol % of **[Ru]-7**, a nearly quantitative conversion of unsaturated fatty ester **1** was achievable (Table 2, entry 4). Moreover, the undesired self-metathesis products from **1** were obtained in lower amounts (ca. 14% of each self-metathesis product **5** and **6**). From the results, it can be concluded that a catalyst loading of above 1.0 mol % of the ruthenium catalyst **[Ru]-7** was necessary for efficient conversion of *cis*-2-butene-1,4-diyl diacetate (**2**). Further increasing the quantity of **[Ru]-7** led neither to an essentially higher oleochemical conversion nor to higher cross-metathesis yields (Table 2, entry 5). The yields of self-metathesis products also remained constant (about 15%).

**Table 2:** Results of catalytic investigations of cross-metathesis of **1** with **2** with various **[Ru]-7** loadings.^a^

entry	**[Ru]-7** loading [mol %]^b^	conversion **2** [%]^b^	yield **3** [%]^b^	yield **4** [%]^b^	yield **5** [%]^b^	yield **6** [%]^b^

**1**	0.1	15	1	2	7	7
**2**	0.5	32	6	6	12	13
**3**	1.0	90	59	58	15	16
**4**	1.5	94	66	65	15	14
**5**	2.0	96	64	63	15	15

^a^reaction conditions: **[Ru]-7**, *m*(**1**) = 0.17 mmol, 0.85 mmol **2**, *n*(PhSiCl_3_)/*n*(**[Ru]-7**) = 100/1, toluene, 5 h, 50 °C, 900 rpm; ^b^determined by gas chromatography with internal standard.

The ratio of the two cross-metathesis reaction partners **1** and **2** has also a great influence on conversion (Table 3). Besides, it is also advantageous to reduce the excess of protected diol **2** in terms of green chemistry and industrial implementation.

**Table 3:** Results of catalytic investigations of cross-metathesis of **1** with various amounts of **2**.^a^

entry	equiv of **2**	conversion **2** [%]^b^	yield **3** [%]^b^	yield **4** [%]^b^	yield **5** [%]^b^	yield **6** [%]^b^

**1**	2	96	45	45	25	26
**2**	4	92	54	54	19	18
**3**	5	94	66	65	13	14
**4**	8	96	76	78	10	9
**5**	10	96	77	79	10	10

^a^reaction conditions: 1.5 mol % **[Ru]-7**, *m*(**1**) = 0.17 mmol, *n*(PhSiCl_3_)/*n*(**[Ru]-7**) = 100/1, toluene, 5 h, 50 °C, 900 rpm; ^b^determined by gas chromatography with internal standard.

Independent of the excess of **2** used, the conversion of methyl oleate (**1**) was quite high (<96%). The cross-metathesis yields reached its maximum at 77% using an eightfold excess of **2** (Table 3, entry 4). The yields could be increased by 32% to 77%. This indicates that the self-metathesis reaction was more and more suppressed; the yields of **5** and **6** were decreased by 15% to 10%. Further investigations were performed with an eightfold excess of **2**, because an additional excess of **2** did not have a positive effect on conversions and yields (Table 3, entry 5). Too high an excess of **2** hindered the conversion of methyl oleate (**1**).

The reactions were stopped after fixed reaction times in an attempt to shorten the necessary reaction time (Table 4). After 1 h, 94% of methyl oleate (**1**) was already converted (Table 4, entry 1). The highest yields of each cross-metathesis product **3** and **4** were just obtained after 5 h (Table 4, entry 3). With longer reaction times, conversions and yields remained constant. The reaction equilibrium was shifted towards the desired cross-metathesis products **3** and **4**, whereas the self-metathesis reaction of **1** was more and more suppressed and the yields of **5** and **6** amounted to around 10%.

**Table 4:** Results of variation of the reaction time of cross-metathesis of **1** with **2**.^a^

entry	time [h]	conversion **2** [%]^b^	yield **3** [%]^b^	yield **4** [%]^b^	yield **5** [%]^b^	yield **6** [%]^b^

**1**	1	94	58	61	18	17
**2**	3	94	67	71	13	14
**3**	5	96	78	82	9	8
**4**	7	93	73	78	10	10
**5**	9	93	70	71	11	10

^a^reaction conditions: 1.5 mol % **[Ru]-7**, *m*(**1**) = 0.17 mmol, 1.36 mmol **2**, *n*(PhSiCl_3_)/*n*(**[Ru]-7**) = 100/1, toluene, 50 °C, 900 rpm; ^b^determined by gas chromatography with internal standard.

Moreover, the conversion of methyl oleate (**1**) appears to be temperature-independent (Table 5); conversions were always higher than 92%. The unsaturated methyl oleate (**1**) underwent a rapid self-metathesis at low reaction temperatures (Table 5, entry 1). In contrast, the cross-metathesis became more predominant at higher reaction temperatures. This suggests that thermal activation of the *cis*-2-butene-1,4-diyl diacetate (**2**) is required. On increasing the reaction temperature from 30 to 50 °C an increase in the yields of the cross-metathesis products (up to 77%) was observed. At the same time the self-metathesis reaction of **1** was hindered and only 10% of each of the self-metathesis products **5** and **6** was produced.

**Table 5:** Results of variation of the reaction temperature of cross-metathesis of **1** with **2**.^a^

entry	temperature [°C]	conversion **2** [%]^b^	yield **3** [%]^b^	yield **4** [%]^b^	yield **5** [%]^b^	yield **6** [%]^b^

**1**	30	92	44	45	24	23
**2**	40	94	55	53	20	19
**3**	50	96	76	78	10	9

^a^reaction conditions: 1.5 mol % **[Ru]-7**, *m*(**1**) = 0.17 mmol, 1.36 mmol **2**, *n*(PhSiCl_3_)/*n*(**[Ru]-7**) = 100/1, toluene, 5 h, 900 rpm; ^b^determined by gas chromatography with internal standard.

Finally, it was desirable to avoid the use of protecting groups. Thus, the optimised reaction conditions for the cross-metathesis of methyl oleate (**1**) with *cis*-2-butene-1,4-diyl diacetate (**2**) were applied to the cross-metathesis reaction of the corresponding fatty acid **7** with the diol **8**. The oleic acid (**7**) was reacted with both *cis*-2-butene-1,4-diyl diacetate (**2**) and the diol **8** (Table 6).

**Table 6:** Influence of the protecting groups on the ruthenium catalysed cross-metathesis.^a^

entry	cross-metathesis		c(**[Ru]-7**) [mol %]	X(**1** or **7**) [%]	Y(*α,ω*-product) [%]

	substrate	co-substrate			

**1**	**1**	**2**	1.5	96	78
**2**	**7**	**2**	3.0	75	55
**3**	**7**	**8**	4.0	76	53

^a^reaction conditions: **[Ru]-7**, *m*(**1** or **7**) = 0.17 mmol, *n*(**1** bzw. **7**)/*n*(**2** bzw. **8**) = 1/8, *n*(PhSiCl_3_)/*n*(**[Ru]-7**) = 100/1, toluene, 5 h, 50 °C, 900 rpm.

Whilst the conversion of methyl oleate (**1**) with the protected diol **2** was nearly quantitative using 1.5 mol % of the ruthenium complex **[Ru]-7** at 50 °C within 5 h (Table 6, entry 1), comparative results in the cross-metathesis of oleic acid (**7**) with **2** were only achieved with the use of 3.0 mol % of the same ruthenium catalyst. Under otherwise similar reactions conditions, 75% of oleic acid was converted (Table 6, entry 2). The cross-metathesis yield amounted to 55%. In the complete absence of protecting groups, a catalyst loading of 4.0 mol % was necessary to produce similar results (Table 6, entry 3). With regard to technical implementation, it seems to be more economical to use the protected substrates, since the catalyst loading of the expensive ruthenium complexes is considerably higher.

## Conclusion

In conclusion, the cross-metathesis of methyl oleate (**1**) with *cis*-2-butene-1,4-diyl diacetate (**2**) was feasible with the relatively low catalyst loading of the Schiff base ruthenium catalyst **[Ru]-7** to yield two value-added and sustainable intermediates in one step. Methyl 11-acetoxyundec-9-enoate (**3**) and undec-2-enyl acetate (**4**) are both very interesting substrates for polymer synthesis. They could be prepared under mild reaction conditions within 5 h. Moreover, this is an advantageous contribution towards the synthesis of sustainable monomer units because a new *α*,*ω*-difunctional substrate class starting from a renewable compound and an inexpensive base chemical was prepared.

Various metathesis catalysts were investigated, disclosing that the Schiff base ruthenium indenylidene catalyst **[Ru]-7** bearing a *N*-heterocyclic carbene ligand, which is an already industrial implemented metathesis catalyst, led to high conversions and yields of the desired cross-metathesis products. Interestingly, this cross-metathesis could be performed without protecting groups, but the catalyst loading had to be adjusted to get similar oleochemical conversions and cross-metathesis yields.

## Experimental

### Materials

Sunflower oil with a high oleic content (91.4% oleic acid) was obtained from Emery Oleochemicals. *cis*-2-Butene-1,4-diol (**8**) (97%), solvents and reagents were purchased from Sigma-Aldrich. Benzylidene ruthenium catalysts **[Ru]-1** and **[Ru]-2** were obtained from Sigma-Aldrich and the remaining indenylidene ruthenium catalysts **[Ru]-3**–**[Ru]-8** were provided by *Umicore* AG & Co. KG and were used as received.

All reactions were performed under an inert atmosphere of argon using standard Schlenk line techniques. Methyl oleate (**1**) was prepared by transesterifcation of high oleic sunflower oil with methanol using hypostoichiometric amounts of sodium methoxide (30% in methanol). *cis*-2-Butene-1,4-diyl diacetate (**2**) was prepared by the pyridine catalysed acylation of the diol **8** with acetic anhydride according to [32].

### Analytical equipment and methods

Analytical thin-layer chromatography (TLC) was performed on silica gel TLC-cards (layer thickness 0.20 μm, VWR International). Substrates were visualised with *p*-anisaldehyde reagent. Flash chromatography was conducted on silica gel 60 (40–60 μm, Acros Organics). Nuclear magnetic resonance (NMR) spectra were recorded in deuterated chloroform on a Bruker AVANCE DRX spectrometer operating at 400 MHz at 298 K. Chemical shifts (δ) are indicated in parts per million relative to tetramethylsilane as internal standard (TMS, δ = 0.0 ppm). Gas chromatographic (GC) analyses were performed on a Hewlett-Packard HP 6890 apparatus equipped with a HP5 capillary column (coating: 5% diphenyl-95%-dimethyl-polysiloxane; length 30 m, diameter 0.25 mm, thickness 0.25 μm) and flame ionisation detection (FID) connected to an autosampler. The oven temperature program was as follows: initial temperature 130 °C, hold for 6 min, increase by 25 °C/min to 320 °C, hold for 4 min. Measurements were performed in split–split mode (split ratio 70:1) using nitrogen as the carrier gas (linear velocity of 30.0 cm/s at 300 °C). Conversions and yields were determined with *n*-pentadecane as internal standard and isopropyl alcohol as solvent.

GC–mass spectroscopy (MS) chromatograms were recorded using a Hewlett-Packard HP 6890 instrument with the same capillary column as specified above and a HP 5973 mass detector set (70 eV). The oven temperature program, the split–split mode and the specifications of the carrier gas were similar to those in the GC-FID method.

### Cross-metathesis of methyl oleate (**1**) and *cis*-2-butene-1,4-diyl diacetate (**2**)

A flame-dried Schlenk tube was charged with 0.050 g (0.17 mmol) methyl oleate (**1**) and 2–10 equiv *cis*-2-butene-1,4-diyl diacetate (**2**). The mixture was diluted to 1.250 g with toluene. The solid metathesis catalysts **[Ru]-1**–**[Ru]-8** were added in the range of 0.1–2.0 mol % to the reaction mixture. In the case of Schiff base ruthenium catalysts **[Ru]-5**–**[Ru]-8**, 100 equiv of phenyltrichlorosilane (relative to the catalyst) were also added. The reaction mixture was stirred magnetically at the appropriate temperature (20–50 °C) for the appropriate time (1–9 h). After completion of the reaction, the mixture was cooled to ambient temperature. Conversion and yield analyses were performed by gas chromatography. The metathesis products were isolated after removing toluene in vacuo by flash chromatography on silica gel with cyclohexane/ethyl acetate (from 10/1 to 1/2) as eluent, and subsequently characterised by NMR spectroscopy.

### Characterisation of the substrates

#### Methyl oleate (**1**)

^1^H NMR (400 MHz; CDCl_3_): δ (ppm) = 0.88 (t, 3H, *J* = 8.0 Hz, -CH_3_), 1.28 (m, 20H, -CH_2_-), 1.61 (m, 2H, -C(O)-CH_2_-CH_2_-), 2.00 (m, 4H, -CH_2_-CH=), 2.30 (t, 2H, *J* = 8.0 Hz, -C(O)-CH_2_-), 3.66 (s, 3H, -CH_3_), 5.34 (m, 2H, -CH=CH-). ^13^C NMR (100 MHz; CDCl_3_): δ (ppm) = 14.0, 22.6, 24.9, 27.0, 27.1, 28.9, 29.0, 29.1, 29.2, 29.4, 29.6, 29.7, 31.8, 34.0, 51.3, 129.6, 129.9, 174.2. MS (EI, 70 eV): *m/z* (%) = 296 (4) [M^+^], 264 (28), 246 (2), 235 (3), 222 (17), 207 (3), 194 (3), 180 (16), 166 (8), 152 (10), 137 (14), 123 (24), 110 (29), 97 (54), 83 (55), 74 (65), 69 (68), 55 (100), 41 (76), 29 (29).

#### *cis*-2-Butene-1,4-diyl diacetate (**2**)

^1^H NMR (400 MHz; CDCl_3_): δ (ppm) = 1.96 (s, 6H, -C(O)-CH_3_), 4.57 (d, 4H, *J* = 4.0 Hz, -O-CH_2_-), 5.64 (m, 2H, -CH=). ^13^C-NMR (100 MHz; CDCl_3_): δ (ppm) = 20.6, 59.7, 127.8, 170.3. MS (EI, 70 eV): *m/z* (%) = 172 (14) [M^+^], 113 (7), 99 (1), 82 (2), 70 (46), 61 (4), 53 (2), 43 (100), 39 (6), 27 (6).

#### Methyl 11-acetoxyundec-9-enoate (**3**)

^1^H NMR (400 MHz; CDCl_3_): δ (ppm) = 1.61 (m, 8H, -CH_2_-), 1.66 (m, 2H, -C(O)-CH_2_-CH_2_-), 2.05 (s, 3H, -C(O)-CH_3_), 2.07 (m, 2H, =CH-CH_2_-), 2.30 (m, 2H, -C(O)-CH_2_-), 3.66 (s, 3H, -O-CH_3_), 4.67 (d, 2H, *J* = 8.0 Hz, -O-CH_2_-CH=), 5.87 (m, 2H, -CH=). ^13^C NMR (100 MHz; CDCl_3_): δ (ppm) = 21.0, 24.9, 28.7, 29.0, 29.3, 29.7, 32.2, 51.4, 65.3, 129.7, 130,9, 170.9, 174.2. MS (EI, 70 eV): *m/z* (%) = 256 (2) [M^+^], 213 (3), 196 (3), 182 (23), 164 (31), 154 (8), 135 (14), 122 (14), 107 (8), 98 (15), 81 (29), 67 (24), 55 (34), 43 (100), 29 (9).

#### Undec-2-enyl acetate (**4**)

^1^H NMR (400 MHz; CDCl_3_): δ (ppm) = 0.88 (t, *J* = 8.0 Hz, 3H, -CH_3_), 1.29 (m, 12H, -CH_2_-), 2.56 (m, 5H, -CH_2_-CH=, -CH_3_), 4.50 (d, *J* = 8.0 Hz, 2H, -O-CH_2_-CH=), 5.66 (m, 2H, =CH-). ^13^C NMR (100 MHz; CDCl_3_): δ (ppm) = 11.4, 21.0, 22.7, 29.2, 29.2, 29.3, 29.4, 31.9, 32.2, 65.3, 129.8, 136.8, 179.3. MS (EI, 70 eV): *m/z* (%) = 212 (4) [M^+^], 170 (5), 152 (3), 141 (4), 124 (12), 110 (8), 96 (19), 82 (26), 67 (25), 54 (31), 43 (100), 39 (11), 29 (14).

#### 9-Octadecene (**5**)

^1^H NMR (500 MHz; CDCl_3_): δ (ppm) = 0.89 (m, 6H, -CH_3_), 1.28 (s, 24H, -CH_2_-), 2.00 (m, 4H, =CH-CH_2_-), 5.37 (m, 2H; =CH-). ^13^C NMR (125 MHz; CDCl_3_): δ (ppm) = 13.1, 25.9, 26.2, 28.2, 28.5, 28.8, 31.0, 33.4, 129.4. MS (EI, 70 eV): *m/z* (%) = 252 (5) [M^+^], 154 (1), 139 (2), 125 (10), 111 (29), 97 (59), 91 (1), 83 (68), 79 (7), 69 (79), 65 (4).

#### Dimethyl octadec-9-enedioate (**6**)

^1^H NMR (500 MHz; CDCl_3_): δ (ppm) = 1.27 (m 16H, -CH_2_-), 1.59 (m, 4H, -C(O)-CH_2_-CH_2_-), 1.93 (m, 4H, =CH-CH_2_-), 2.26 (m, 4H, -C(O)-CH_2_-), 3.63 (s, 6H, -O-CH_3_), 5.34 (m, 2H, =CH-). ^13^C NMR (125 MHz; CDCl_3_): δ (ppm) = 24.9, 28.9, 29.4, 29.5, 29.6, 32.5, 34.0, 51.3, 130.2, 174.2. MS (EI, 70 eV): *m/z* (%) = 340 (1) [M^+^], 308 (7), 290 (3), 276 (16), 265 (1), 207 (1), 165 (7), 151 (11), 133 (12), 121 (13), 109 (18), 95 (38), 81 (59), 74 (44), 67 (58), 55 (100).
